# Are Microplastics a Macro Issue? A Review on the Sources of Contamination, Analytical Challenges and Impact on Human Health of Microplastics in Food

**DOI:** 10.3390/foods12213915

**Published:** 2023-10-25

**Authors:** Cristina Di Fiore, Fabiana Carriera, Mario Vincenzo Russo, Pasquale Avino

**Affiliations:** 1 Department of Agricultural, Environmental and Food Sciences (DiAAA), University of Molise, 86100 Campobasso, Italy; fabiana.carriera@unicas.it (F.C.); mvrusso@unimol.it (M.V.R.); avino@unimol.it (P.A.); 2 Department of Civil and Mechanical Engineering, University of Cassino and Southern Lazio, 03043 Cassino, Italy; 3 Institute of Atmospheric Pollution Research, Division of Rome, c/o Ministry of Environment and Energy Security, 00147 Rome, Italy

**Keywords:** microplastics, food, beverages, analytical approaches, human health

## Abstract

In recent years, human populations’ exposure to microplastics via foods is becoming a topic of concern. Although microplastics have been defined as “emerging contaminants”, their occurrence in the environment and food is quite dated. This systematic review aims to investigate the discrepancies which are characterizing the research in the microplastics field in foods, with particular regard to sample preparations, microplastics’ concentrations and their effect on humans. For the selection of papers, the PRISMA methodology was followed. Discrepancies in the methodological approaches emerged and in the expression of the results as well, underlying the urgency in the harmonization of the methodological approaches. Uncertainties are still present regarding the adverse effects of microplastics on the human body. The scientific evidence obtained thus far is, in fact, not sufficient to demonstrate a concrete negative effect. This review has clearly underlined the need to standardise laboratory approaches to obtain useful results for better food safety management.

## 1. Introduction

The occurrence of microplastics in food products is becoming a topic of public concern. Ingestion, together with inhalation and skin perfusions, represents the main human intake routes of microplastics [1]. Although various food products have been found to be contaminated with microplastics, knowledge for many other foods is still lacking. For instance, food products such as drinking water [1,2,3,4], seafood [5,6,7,8,9,10,11,12,13,14], honey [15,16] sea salt [17,18,19,20,21,22,23,24,25,26,27,28], beer [16,29] and sugar [18,30] are the most investigated thus far. On the contrary, food products such as tea [31], packaged chicken meat [32,33] and milk products [34] are poorly studied. Food matrices are exposed to a risk of microplastics contamination. The latest findings have suggested that both food processing and packaging are sources of microplastics [35]. Furthermore, modifications in food processing, transportation and storage conditions are likely to change the content of microplastics in foods [36]. However, the lack of sensitive and standardized detection, as well as the pre-treatment of food samples, does not allow us to deeply assess the presence of microplastics in food. Furthermore, the multidimensionality of the data necessary to clearly describe the contamination by microplastics in food products is still challenging to be managed by researchers. As a consequence, the validation of analytical procedures is still far from being achieved. Furthermore, researchers have adopted various analytical approaches and procedures (i.e., from the preparation of the food sample to the chemical analyses) [10]. Hence, the comparison of data among results from different studies is challenging. Likewise, the absence of validated methodologies for microplastic detection in foods and beverages determines the application of inappropriate analytical methodologies [37]. Furthermore, the prevention of airborne contamination during laboratory experiments is still challenging [4]. For instance, a work by Lachenmeier et al. (2015) [37] reported that a specialized cleanroom cannot totally avoid airborne contamination because cleanroom classifications focus on small particles and may exclude the relevant sizes of microplastic particles [37]. Food products are a vector for microplastics to enter into the human body and potentially produce adverse effects [38]. Microplastics, ranging in size from 50 to 500 μm, have been detected in the human stool of healthy people exposed to microplastics via foods [39]. Up to now, it is known that microplastic particles may cause abrasions or blockages of the gastro-intestinal system of animals, as well as small microplastics which can translocate into body tissues, determining adverse effects. Furthermore, the smallest microplastics (<1.5 μm) can potentially penetrate into organs [4]. However, the size range of microplastic particles that is capable of being embedded in tissues should be clarified.

The present review aims to highlight the gaps present in the available scientific literature on microplastics and food. In particular, the review will seek to (i) report the concentrations at which microplastics are present in the investigated food matrices thus far; (ii) identify the gaps in the preparative methodological approaches to chemical analysis; (iii) identify the gaps related to the potential harmful effects on the human body in correlation with the physical characteristics (i.e., size) of microplastics. To the best of our knowledge, this is the first review underlying the importance of the application of the same analytical protocol starting from the preparation of the samples, as well as the need to deepen knowledge of the adverse effects on the human organism in relation to the size of microplastics.

## 2. Materials and Methods

**Figure 1:**
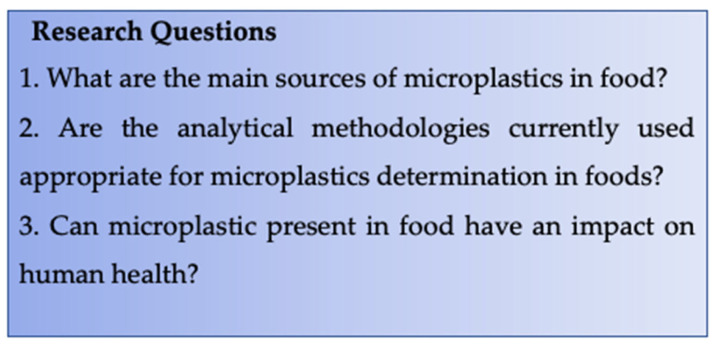
Research questions used for conducting the research of articles.

This review was designed systematically, following the PRISMA guidelines [40]. Firstly, the main concepts concerning microplastics discussed in this review were identified by formulating the “Research questions” (Figure 1).

Specifically, microplastics’ occurrence in foods, analytical approaches (i.e., sample preparation and chemical identification of microplastics) and the negative and adverse impact on humans were considered. Therefore, search terms for each concept were identified and used for searching the relevant articles on Google Scholar and Scopus databases. The terms belonging to each concept were combined using the Boolean operator “OR”, whereas the categories were then combined using the Boolean operator “AND” (Table 1).

All articles obtained were screened in a two-step process. The first step was based on the selection of articles (i.e., only original studies published in a peer-reviewed journal) by analysing the title and the abstracts. Articles which did not focus on microplastics in foods were excluded.

The second step was carried out by reading the full text. The selection of articles was performed following the inclusion and exclusion criteria. The inclusion criteria were to include in the revision only articles reporting the presence of microplastics in food matrices (as well as sources of contamination) with a focus on human health implications, limitations in microplastic’s analysis and in sample preparation. Therefore, the articles included presented information about the presence of microplastics in foods and beverages, including the potential sources of contamination. Likewise, articles that present relevant information for the identification of gaps in the analytical methodologies were considered in this review. Finally, articles which include information on the interaction between microplastic particles and humans after ingestion were included.

## 3. Results

**Figure 2:**
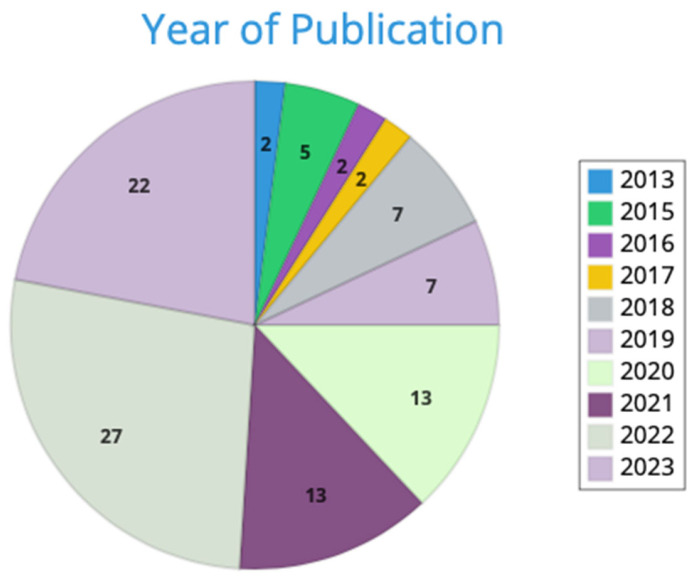
The proportion (%) of included articles by year of publication.

The research of papers by means of search terms identified in the Scopus and PubMed databases returned a total of 1795 papers (net of duplicates). Therefore, 856 articles were excluded through the title and abstract screening. The remaining 939 papers were screened by reading their full text. A total of 64 papers remained and were included in the present review. In total, 27.7% of papers included were published in 2022, whereas 21.7% were published in 2023 (Figure 2).

**Figure 3:**
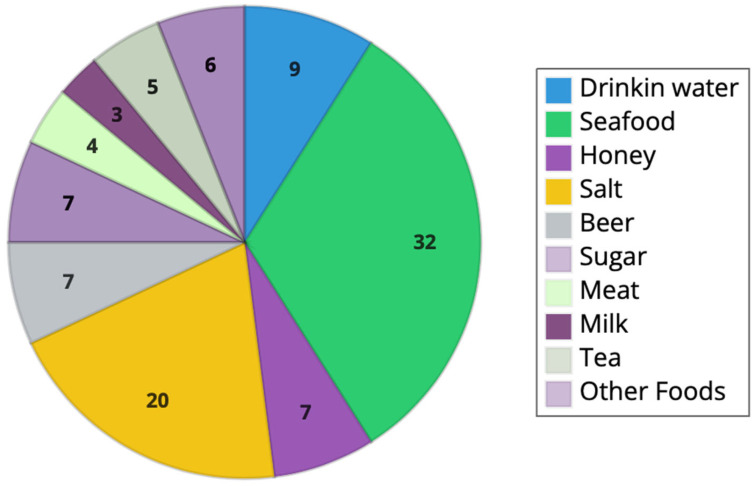
The proportion (%) of included articles by food matrix analysed.

In total, 32% of the included papers were about the investigation of microplastics in fish food, whereas 20% were about sea salt’s analysis; 9% were focused on drinking water; 7% each were about honey, beer and sugar; 3% were about milk; 4% about meat; 5% about tea; and 6% about other foods (Figure 3).

### 3.1. Main Sources of Microplastics in Food and Beverages

The detection of sources of microplastics in food is very complex. Scientific literature analysis shows that the main sources of microplastics in food are the production process, contamination of raw materials for processed food products [1] or beverages and packaging [1,16].

#### 3.1.1. Mineral Bottled Water

Microplastic contamination of mineral drinking water stored in plastic bottles has been reported. Works by Zuccarello et al. (2019) [1] and Samandra et al. (2022) [2] reported the presence of microplastic spheres of polyethylene terephthalate (i.e., PET) (1.28 and 4.2 μm) and particles of polypropylene, polyamide and polyethylene (i.e., PP, PA and PE) (77 ± 22 μm) in a concentration of 656.8 μg L^−1^ ± 632.9 μg L^−1^ and 13 ± 19 microplastics L^−1^ in mineral bottled water samples, respectively [1,2]. Some evidence seems to suggest a contribution by the packaging: bottle and caps. Concerning the caps, Samandra et al. (2022) [2] suggested the potential contribution of the twistable and push-top caps, but no experimental evidence resulted [2]. Instead, the bottle capping process has been suggested as a potential microplastics source. For instance, laboratory tests conducted by Weisser et al. (2021) [3] showed an increase in microplastic concentration from 1 microplastic L^−1^ to 317 ± 257 microplastics L^−1^ (11–500 μm) due to the capping of drinking water bottles [3]. Furthermore, cap sealing materials have a low abrasion resistance to mechanical stress, leading to a release of microplastics in the water [3,41]. In the case of bottles, mineral water stored in single-use and reusable bottles made of PET showed 2649 ± 2857 microplastics L^−1^ and 4889 ± 5432 microplastics L^−1^ (<5 μm), respectively [4]. Plastic bottles, in particular reusable bottles, can be subjected to stresses (e.g., washing process) that can influence microplastic particles’ release [4]. Microplastic particles have been found in mineral water stored in glass bottles. Particularly, a concentration of 6296 ± 10,521 microplastic L^−1^, made of PE, PP, styrene-butadiene-copolymer (i.e., SBC) and polystyrene (i.e., PS) (95% were than <5 μm; 5% were <1.5 μm) has been determined. PP and PE can be due to the cap itself, whereas SBC and PS cannot be explained by the packaging release. Therefore, other sources of contamination should be taken into account [4]. Firstly, caps are generally stored in open containers. This leads to a remarkable contamination from air [3,41]. Furthermore, glass bottles are washed more intensively than plastic ones. Therefore, the washing liquid should be contaminated by microplastics, as well as some machine parts used for washing releasing microplastics [4]. Therefore, the main sources of microplastic particles in mineral bottled water are the packaging (bottle and caps), capping bottle process, air and, particularly for water stored in glass bottles, the washing machine parts and liquid.

#### 3.1.2. Fish Products

Microplastics have been detected in industrial fish meal. Particularly, PET have been quantified at 12.9 mg kg^−1^. On the other hand, contamination by PS has been hypothesized, but no confirmation has been achieved due to sensitivity limitations of the pyrolysis-gas chromatography-mass spectrometry (i.e., Py-GC/MS) [42]. PP microplastics (1.2 to 10 µm) have also been revealed in edible muscles of swordfish and bluefin tuna in concentrations of 140 to 270 and 160 to 270 number of microplastic particles kg^−1^ [43]. Fibrous microplastics (0.06 to 0.1 mm) have been detected in different species of wild-caught seafood. The concentrations revealed ranged from 0.04 to 1.8 microplastics g^−1^ of tissues [44]. Gastrointestinal tracts of edible fishes showed fibrous and fragments microplastics ranged from 109 to 284 µm. The mean concentrations detected were of 128 and 187 microplastics per individual [45]. Contamination of seafood has been affecting shellfish as well. Mean concentration found was about 0.3 to 5.76 microplastics g^−1^ of tissues [46,47]. Bivalves, such as mussels, are known to be highly contaminated with microplastics due to their filtering behaviour, which leads to the ingestion of significant amounts of seawater and microplastics. In a study conducted by Dambrosio et al. (2023) [48], it was found that mussels contained an average of 1.59 ± 0.95 MPs g^−1^ and 6.51 ± 4.32 MPs individual^−1^. Blue fragments, sized 10–500 μm, were the prevalent findings; most of them belonged to PA polymers [48]. Several commercially important crustacean species, including brown shrimp and tiger prawn, have been discovered to contain ingested microplastic particles. Devriese et al. (2015) [49] found an average of 0.68 ± 0.55 particles/g in brown shrimp, whereas Abbasi et al. (2018) [50] detected 1.5 particles/g in tiger prawns. Microplastics have also been detected in different species of dried fish products. Kutralam-Muniasamy et al. 2023 [51] discovered that two commercially important *Chirostoma* species (*C. jordani* and *C. patzcuaro*) in Mexico contained a varying range from 4.00 ± 0.94 to 55.33 ± 9.43 particles g^−1^. Rukmangada et al. 2023 [52] detected 99 ± 18.91 MPs g^−1^ from a sample of *Anguilla bengalensis* [52].

#### 3.1.3. Sea Salt

The scientific literature has reported microplastic contamination of sea salt. For example, brands of Iranian table salts showed an average concentration of microplastics of 151 ± 61.8 microplastic kg^−1^, where 4% were identified as fibres and 96% as fragments. Microplastic particles were identified as PE and PP. With regard to the size, 59% of microplastics were of 1000–5000 μm, whereas 18% and 23% of the remaining microplastics were in the size ranges of 500–1000 and <500 μm. Considering the colour, it resulted that the majority of microplastics were white, followed by black, red, blue and green. Therefore, it was plausible to suggest that the microplastics found originated from different sources [18]. Likewise, a work by Di Fiore et al. (2023) [17] analysed the contamination of sea salt obtained from three Italian salterns. A total of 1653 ± 29 microplastic kg^−1^ was found, confirmed by micro-attenuated total reflectance infrared spectroscopy (i.e., μ-ATR-FTIR) and Raman spectroscopy. In total, 80.6% of the microplastics identified were classified as fibres, 18.9% as fragments and 0.5% as spheres, with the most frequent size being between 0 and 500 μm. The polymers identified were PP, PA and PE [17]. Similar results emerged from a paper of Nakat et al. (2023) [19] who investigated the contamination of Lebanese sea salts available on the market. The average concentration of microplastics was between 0 and 114 microplastic kg^−1^. In total, 35.3% of microplastics were PE and 11.8% PP, assessed by means of μ-FTIR. However, only particles > 100 μm was confirmed to be microplastics by μ-FTIR because of its technical limitations [19]. A work by Manimozhi et al. (2022) [20] investigated the occurrence and abundance of microplastics in India. The maximum occurrence of microplastics was of 52 microplastic kg^−1^. In total, 40% of microplastics showed a size < 100 μm and the majority were identified as fragments [20]. Likewise, sea salts collected from India showed an average concentration of 35 ± 15–72 ± 40 microplastics kg^−1^, where the most common polymer types were PE (51.6%), PP (25%) and polysulfone (i.e., PES) (21.8%), confirmed by μ-ATR-FTIR. PE microplastics were identified as fibres, with sizes ranging from 100 to 500 μm [27]. Commercial sea salt from Sri Lanka showed a contamination ranged within 17 ± 5.9–122.5 ± 64.2 microplastics kg^−1^. Low-density polyethylene (i.e., LDPE) (17%) and high-density polyethylene (i.e., HDPE) (15%) were the most common types of polymers. With regard to the size, 50% of microplastics ranged from 2500 to 100 μm [21]. Likewise, sea salts collected from Europe showed a contamination ranging from 74 ± 105 to 1155 ± 140 microplastics kg^−1^ where 75.6% were identified as fibres and 24.4% as fragments/sheets. The majority of fibres were <155 μm in diameter and 80.1% of fragments/sheets were <155 μm in length. Microplastics were confirmed by using μ-FTIR as rayon, PP, polyester (i.e., PES) and PE [23]. Commercial Australian sea salts were revealed to be contaminated by microplastics. For instance, a work by Kuttykattil et al. (2023) [24] reported an average contamination of 85.2 ± 63.0 microplastic kg^−1^. μ-ATR-FTIR analyses identified PET (5.6%), PES (2.8%), PE (2,8%), PP (8.5%), cellulose acetate (i.e., AC) (4.2%), polyurethane (i.e., PU), (30.9%), PA (33.8%) and polyvinyl chloride (i.e., PVC) (2.8%). With regard to the shape, fibres and fragments contributed 75.8 and 24.2%, respectively. The size range of microplastics was between 23.3 μm and 3.9 mm [24]. The contamination of sea salts of different origins showed a contamination around 700 microplastics kg^−1^, and the size was within 5.2 mm to 3.8 μm. FTIR identified cellophane (CP), PS, PA and polyarylether (i.e., PAR) as the most common polymer types [26]. Spanish sea salt has been documented to be affected by microplastics as well. In total, 50–280 microplastic kg^−1^, being mainly PET, were detected in Spanish sea salt. Fibre was the predominate shape, with size ranging from 30 μm to 3.5 mm [28]. The presence of microplastics in table sea salt might originate from various sources. Firstly, the significant background presence of microplastics in the environment, especially marine ones, can represent one of the main sources of microplastics in sea salt [28]. As technologies applied to sea salt, such as the packaging process, cannot influence microplastics abundance [17], they should come from mainly the environment [19]. For instance, Sivagami et al. (2021) [26] suggested that the occurrence of cellophane (i.e., CP) in sea salt is due to the increase in the use of CP by food industries, contributing to the release of cellophane microplastics into the environment [26]. In contrast, Makhdoumi et al. (2023) [18] suggested the packaging contribution to the microplastics contamination of sea salts. In Iran, sea salt is commonly stored in plastic packaging. Therefore, opening and closing the containers might release microplastics into the salt [18]. However, scientific literature reported the presence of microplastics in raw sea salt worldwide (i.e., 1000–2000, 620–1200, 36–3345, 470–1633, 2395, 13,500–19,800 microplastics kg^−1^), meaning that the contamination occurred before the sea salt processing [22].

#### 3.1.4. Honey

Honey contamination by microplastic particles has thus far not been documented. For example, a work by Liebezeit et al. (2014) [53] showed synthetic particles contamination of honeys from Germany and Italy. Particularly, fragments and fibres were detected, with a length ranging within 40 μm–9 mm and 10–20 μm, respectively. The concentration of fibres detected was between 20 and 330 synthetic particles per 500 g of honey, whereas fragments were present in a concentration of 0–19 synthetic particles per 500 g of honey. However, the synthetic particles were not confirmed as plastic [53]. More recently, Alma et al. (2023) [15] investigated microplastics contamination of honey, identifying the environment as the main source. Microplastic particles present in the atmosphere can in fact adhere to pollen due to a sticky substance (i.e., pollenkitt). Therefore, honeybees can inadvertently transfer microplastics to the hive [15,53]. This has been confirmed by experimental tests. About 670 microplastics kg^−1^ have been transferred to the hive by honeybees [15]. Honey processing can contaminate honey itself. For instance, the workup of honeycombs can release microplastics, as well as cloths soaked with formic or oxalic acid used by beekeepers to fight infesting beehives. Moreover, plastic bags used to supply powdered sugar to the bees in particular times can be considered as another source of microplastics [53]. Honeys produced using different raw materials have showed different levels of microplastics contamination. For instance, domestic flower honey, imported flower honey and domestic sugar feeding honey showed a microplastics concentration of 0.33, 0.17 and 0.09 microplastic g^−1^, respectively. The most abundant polymer was PP and 70–100% of microplastics were <300 μm [54]. Likewise, honey collected from supermarkets from different countries showed microplastics concentrations ranging from 40 to 666 microplastics kg^−1^ [15]. Therefore, the sources of microplastic particles in honey cannot be clearly identified. It resulted that the environment and honey processing are the main route for microplastics releasing into honey.

#### 3.1.5. Beer

The occurrence of microplastics in beer samples could likely be derived from packaging. For example, in some countries, beers are stored in PET containers as it is a cheaper packaging material compared to glass. A work by Habschied et al. (2022) [29] suggested microplastics presence in beer samples through the determination of plastic-related compounds [29]. However, PET, PP, PS and PE microplastic fragments have been detected in beer samples by micro-Raman spectroscopy (i.e., μ-Raman) in an average concentration of 20–80 microplastic mL^−1^. Raw materials, containers, as well as airborne microplastics have been suggested as potential sources [55]. For example, craft beer processed in cities with a high population density showed a greater amount of microplastics, meaning that the airborne microplastics can represent a significant source of microplastics in beverages. More specifically, craft beer showed an average microplastic concentration of 29 fibres L^−1^, with a size range within 40.28–769.8 μm. Fragments were detected in a concentration of 240 fragments L^−1^ with a size range within 6.2–128.1 μm [16]. Therefore, even though the occurrence of microplastics in beer samples is scarcely investigated, some sources could be suggested. Packaging, particularly plastic ones, and airborne microplastics can represent two sources of microplastics. Furthermore, water used for beer production could be added to them.

#### 3.1.6. Sugar

Sugar is scarcely documented in microplastic particles contamination. In this regard, a work by Makhdoumi et al. 2023 [18] demonstrated a concentration range between 33 ± 4.2 to 80 ± 4.2 microplastic kg^−1^ with an average of 55.1 ± 43.7 microplastic kg^−1^. Fragments (95.8%) and fibres (4.1%) were determined as the most common shapes, whereas no film, bead or other types of shapes were isolated. With regard to the size, 38% of microplastics had a size ranging between 500 and 1000 μm, 37% were in the 1000–5000 μm size range and 25% were <500 μm. μ-FTIR analysis revealed that microplastic fibres and fragments in sugar were PE. Furthermore, their wide range of colour, such as white or black along with blue and green, suggested that that microplastics originate from several sources [18]. Similarly, microplastic fibres and spheres were detected in sugar both unpacked and packed. A concentration of 343.7 ± 32.08 microplastics kg^−1^ of sugar was found, PVC being the most frequent. The contamination of sugar is due to multiple sources such as processing, refinement and packaging. The stages required to produce crystallized white sugar and refine it into granulated centrifugal sugar are susceptible to the input of MP materials dispersed in industrial environments or present in machinery. Contamination by MPs may be more likely during the sugar drying stage, where poorly operated or undersized dryers can provide an air stream contaminated with MPs, and the passage of sugar through plastic ducts can also be a source [30].

#### 3.1.7. Tea

Teabags seem to be an important source of microplastics. A work by Afrin et al. (2022) [31] conducted a study conducted to investigate the presence of microplastics (MPs) in tea bag samples from different brands. All brand analysed showed fragment and fibre microplastics, with a variety of colours (i.e., brown, blue and red). The size of microplastics found ranged within 200.6 and 220.7 μm, and the identified polymer types included polytetrafluoroethylene (i.e., PTFE), HDPE, polycarbonate (i.e., PC), nylon, PVC, cellulose acetate (i.e., CA) and acrylonitrile butadiene styrene (i.e., ABS) [31]. The sources of microplastics in teabags are the bag itself as the use of polymers in the tea packaging process is widespread among food industries to keep their bags from falling apart. Furthermore, experimental evidence seems to suggest the migration of monomers and oligomers from plastic teabags [31]. In fact, it has been reported that plastic teabags can release billions of microplastics and nanoplastics into a single cup of tea when steeped at a typical brewing temperature of 95 °C [56].

#### 3.1.8. Other Food Products

Even though food matrices such meat and milk products are poorly investigated, some evidence has suggested their affectability by microplastics. For instance, a work by Bilal et al. (2023) [57] investigated the occurrence of MPs in both the crop and gizzard of farm chickens. They collected samples from 24 chickens and found a total of 429 MP particles in the crops, with a mean of 17.8 ± 12.1 MPs/crop. In contrast, 798 MP particles were found in the gizzards, with a mean of 33.25 ± 17.8 MPs/gizzard. Habib et al. (2022) [32], instead, investigated the contamination by microplastics of chicken meat purchased from supermarkets. They reported a concentration of microplastics ranging from 0.03 ± 0.04 to 1.19 ± 0.72 particles g^−1^ of meat. The sizes of microplastics were within 8.2 to 1455 μm, identified as LDPE by differential scanning calorimetry [32]. However, contamination was supposed to be due to the plastic cutting boards on which the meat was cut. On the contrary, meat packaging made of expanded polystyrene (i.e., XPS) released microplastics onto meat. Experimental evidence seems to suggest that a single meat package can release from 4.0 to 18.7 microplastic fragments, confirmed by μ-ATR-FTIR [33].

Milk and related products are not free from microplastic contamination. Farm cows’ milk samples showed a concentration of microplastics ranging from 204 to 1004 microplastics per 100 mL of milk, with a size range within 5 to 40 μm. μ-Raman spectroscopy identified the microplastics as PE, PES, PP, PTFE and PS. However, milk powder and ready-to-drink milk showed relatively higher levels of microplastics (fibre, fragments and beads). Furthermore, it was noticed that the quantity of MPs tended to rise from milk obtained directly from the farm to processed milk powders, despite the fact that the concentrations of MPs remained within the same range [34]. Regarding the sources of contamination, the research indicated that liquid milk samples typically had a low concentration of MPs, with the exception of one raw milk sample obtained from a farm. The primary cause of MP contamination in raw milk is likely to be the presence of PE, a polymer that is used in the milking machine. Other polymers, such as PP, PES and PTFE, found in raw milk may be traced back to various sources throughout the farm environment and milking process, including storage containers. PP was detected in milk samples that had been packaged in bottles, while PE was found in samples that had been stored in multilayer laminated paper packaging [34].

### 3.2. Analytical Challenges in Microplastic’s Determination in Food Samples

The digestion of organic matter is fundamental for microplastics’ assessment in food products [8]. Indeed, as reported, digestion leads to an increase in the number of microplastics extracted from a food matrix (e.g., PS increased from 97 ± 13 to 123 ± 45 in milk samples) [34]. At the same time, though, digestion conditions (i.e., time, temperature and agent type) should guarantee the absence of physico-chemical alterations in the polymer structures [34]. Among scientific studies, there are discrepancies regarding the effects of digestion on microplastics.

Depending on the chemical composition of the food matrix, digestions could require long reaction times (i.e., 12–72 h) [11]. As time is a crucial factor in decision-making and protocol selection, it is essential to choose protocols that adequately address the quantity of microplastics [11]. Experimental approaches for digesting food organic matter are different. Table 2 displays the digestion conditions for various matrices as reported in the scientific literature. When examining edible fish tissues, multiple digestive treatments were identified. The effectiveness of a digestion methodology can be evaluated based on its percentage digestive efficiency (%. DE). However, only two studies [10,58] reported achieved the digestion efficiency necessary. In the case of meat samples, only one study by Habib et al. (2022) [32] proposed an analytical approach, but the effects of combining KOH with high temperature (i.e., 75 °C) have not been investigated. Similarly, inconsistencies exist in the pre-treatment of sea salt. For instance, some papers (*n* = 4) included in this review did not apply digestion pre-treatments to salt samples [17,18,19,28]. On the contrary, seven papers used chemical treatments [20,21,22,23,24,25]. Pre-treatment of beer has been conducted but the direct filtration of the sample appears to have allowed the determination of microplastics as well [29]. Honey, milk and lettuce samples were digested for the degradation of the organic matter. However, no information is provided regarding the temperature applied, the concentration of the reagent and the digestion efficiency.

### 3.3. Microplastic’s Qualification: Techniques’ Limitations

The qualification of microplastics extracted from foods was mainly conducted using spectroscopy techniques (i.e., µ-FTIR, µ-ATR-FTIR, µ-Raman, energy dispersive X-ray Spectroscopy (SEM/EDX)) (Table 3). The size ranges analysed using µ-FTIR and µ-ATR-FTIR were of 3.8 μm–5 mm, 11 μm–5000 μm, 5 μm–5.9 mm, respectively. However, due to the size limitations of the techniques, the qualification of smaller microplastics (<20 µm) using µ-FTIR should be reassessed (see Section 4).

### 3.4. Microplastic’s Impact on Human Health

Humans are exposed to microplastics mainly through foods (i.e., seafood, salt, sugar, honey, drinking water). A rough approximation of microplastics intake via foods and beverages by humans has been estimated to be 48,000 microplastics/year [63]. Instead, according to the EFSA, in the European Union, humans’ intake of microplastics has been estimated at 119 microplastic particles/year [64].

Recently, the World Health Organization underlined the difficulties in the assessment of the potential impact of microplastics on human health. It was reported that the limited availability of data about the health impacts is due to analytical limitations. Particularly, microplastics < 10 µm in size are still complex to detect [65]. Such a determination is relevant for the assessment of the health impact on humans, as smaller microplastics (i.e., <<10 µm) could accumulate in human tissues. Smaller microplastics, in fact, could be able to cross the biological barriers and thus accumulate into cells. Cellular uptake of microplastics has been not investigated in depth. Furthermore, the term “uptake” mostly signifies tissue accumulation, without any consideration to the cellular internalization of microplastics [66]. The studies included in this review did not focus on the mechanisms of microplastics’ embedding in cells’ systems. Microplastics’ size detected in food products in this review seems to be too big (i.e., <1.5–5000 µm) for supposing a cellular uptake. It was reported that this phenomenon is likely biologically possible for nanoscale particles (i.e., 20 nm) [67]. Therefore, the occurrence of microplastics of ~5 µm in drinking water could not be certainly considered as a macro issue. Such considerations could be also confirmed by Shruti et al. (2020). They reported that likely >90% of ingested microplastics (i.e., 50–500 µm) are excreted from the human body [68,69]. Such a size range is, in fact, biologically plausible to be extracted as the sizes are too big to hypothesise a cellular internalisation (see Section 4).

## 4. Discussion

### 4.1. Sources of Microplastics in Food Products and Main Characteristics

Drinking water presents microplastics contamination. The main source of contamination could be the packaging (both bottle and caps). Such contamination is characterised by specific polymer types (i.e., PET, PP), which are commonly used for bottles’ and caps’ production [2,4]. However, water for human consumption comes from different sources (i.e., lakes, river and reservoirs), commonly subjected to microplastics exposure [70]. In fact, the presence of MPs in raw water is influenced by various factors, including the kind of water body and its ambient environment (i.e., presence of human activities and water condition) [71]. Wastewater treatment plants (WWTPs) have demonstrated their capability to partially remove MPs. A study by Zhou et al. (2023) [72] analysed the MPs presence in each treatment unit of a rural WTP in China. During the coagulation and sedimentation treatment, fibrous MPs (most dominant shape found) can agglomerate with each other and large-sized microplastics are captured by flocs and then precipitate. The subsequent sand filtration treatment determines the removal of non-fibrous MPs, which may be related to the polarity of microplastics. Lastly, the ultrafiltration membrane used had an average pore size of 0.02 μm, which was significantly smaller than the size of the microplastics found in the influent. As a result, it was theoretically possible to eliminate the microplastics entirely. However, the MPs abundance slightly increased again when the water was transported to residents’ homes, possible due to the release of MPs from the distribution systems [72].

This finding suggests that the occurrence of polymers, different from PET and PP, in bottled water may have originated from contamination during the production process or from methodological artifacts.

Various types of microplastic are found in sea salt samples. It was suggested that MPs’ abundance in raw salt reflects the level of MP contamination of seawater, as during the production no filtration occurred [21]. Therefore, the main source of microplastics in sea salt is the environment, especially the marine one [28]. However, raw sea salt contains a higher number of microplastics compared to food-grade salt produced in the same saltern [21]. Sea salt is produced by pumping saltwater into evaporation ponds, where it becomes more concentrated because of sunlight and wind. As a result, the salt condenses and forms crystals on the surface of the crystallizers. These crystals are carefully cut and collected using a controlled process. The collected salt then undergoes various physical treatments before being packed into various containers for its diverse uses and applications [28]. The low concentration of MPs in food-grade salt suggested that some MPs are removed due to the cleaning phases of the raw salt; however, no information is reported in the scientific literature. Nevertheless, the process of refining salt is unable to completely remove microplastics (MPs) from the initial raw salt crystals. Additionally, the presence of MPs can persist in salt during both the production and packaging stages, thereby resulting in contamination [73]. As suggested by some researchers, packaging contributes to the presence of some polymer types, such as CP and PP widely used by the food industry [18,26].

The aquatic environment is severely polluted by microplastics. Microplastic pollution in the oceans and seas is mainly caused by plastic waste and activities such as fishing and agriculture. Intense fishing activities often lead to abandoned fleets and boats in coastal waters, thus contributing to the problem of microplastics. Similarly, maritime transport, tourism and other maritime activities can affect the presence of foreign microplastics in aquatic environments [44,45]. In addition to anthropic actions, the pollution of the aquatic environment is also influenced by hydrodynamic aspects such as tidal influences, wave actions and water curvet regimes [44,48]. Aquatic food poses a significant concern due to its vulnerability to contamination by microplastics. In facts, microplastics are ingested by marine life and can ultimately find their way onto consumers’ tables [43]. Mussels are the most exposed to contamination with MPs. “Standard” mussels possess a filtration capacity of 2 L h^−1^, enabling them to filter 24 L of seawater each day during their 12 h filtering period. Considering the average concentration of microplastics (MPs) in seawater (i.e., 0.4 ± 0.3 particles L^−1^), a mussel is estimated to encounter around 10 particles daily [47].

Microplastics are present everywhere in the environment, including the atmosphere, and can have negative effects on living organisms. Microplastic particles present in the air can attach to pollen due to a sticky substance called pollenkitt. Consequently, bees can unknowingly transport microplastics to their hive [15]. In addition, it should be considered that the honey processing process can also contribute to its contamination by microplastics. For example, beekeepers, by handling the honeycombs, can unintentionally release microplastics, as well as the plastic bags used to provide powdered sugar to bees at certain times [53]. It has also been shown that different types of raw materials used in honey production can contain different levels of microplastics [54]. Therefore, it is difficult to clearly identify the sources of microplastics in honey. It has emerged that the environment and the honey processing process represent the main pathways for the release of microplastics in the final product.

It is probable that microplastics found in various food items (such as beer, sugar, tea, etc.) are sourced from the manufacturing process, packaging and airborne microplastics. Additionally, the inclusion of different raw materials and water during production may contribute to their presence [54,55].

### 4.2. Analytical Discrepancies in Food Sample Preparation

The main analytical challenges in the field of microplastics primarily revolve around the extraction and separation of these particles from food samples [34]. The chemical identification of microplastics depends strongly on the successful removal of organic matter [74]. From the analysis of the scientific literature reviewed, an important difference in approaches for the removal of organic matter for the determination of microplastics from food matrices has emerged. Matrices with similar chemical–physical characteristics are treated with different agents, and as a result, undergo different chemical reactions (i.e., saponification, oxidation). Furthermore, the absence (except for *n* = 2 papers) of digestive efficiencies poses a challenge in comparing the results, as well as the use of different times/temperatures which does not allow us to select which analytical approach might be the best. The temperatures used differ among studies, when the same reagent was used (i.e., H_2_O_2_, 30 °C for 24 h, 45 °C for 72 h, 60 °C for 36 h) [12,58,60]. Temperature affects the course of digestion [74]. The increment in temperature should lead to an acceleration of the digestion reaction, but this aspect does not appear to be highlighted in the papers, but this aspect could be considered for the purpose of a more systematic application of digestive methodologies. However, temperature is one of the factors that can determine damage to microplastics [25]. For instance, a work by Habib et al. (2022) [32] used a temperature of 75 °C for digesting meat using KOH (10%) [32]. However, it is suggested that a temperature above 50 °C damages microplastics [41]. Although the literature suggests that temperatures exceeding 50 °C could potentially damage microplastics [75], this paper fails to provide any control or investigation into the impact of digestion on microplastics. Likewise, approaches in microplastics’ determination in sea salt are different. For examples, some papers (*n* = 4) only dissolved sea salt in deionized water, whereas other papers (*n* = 7) used chemical pre-treatments. However, none of the studies suggest an investigation regarding the need to apply a digestion treatment, as well as studies that used chemical pre-treatments. Evaluating the necessity of a digestion treatment for microplastics determination could avoid the application of an acidic, basic or oxidizing treatment, thus reducing the potential impacts on microplastics. Discrepancies have also been identified in the data expression mode. This aspect is evident in water bottles’ analysis. Precisely, microplastics’ concentrations in bottled water are expressed as μg L^−1^ [1] or number of microplastics L^−1^ [2,3,4]. Therefore, the comparison between concentrations is complex.

### 4.3. Microplastic’s Qualification: Techniques’ Limitations

Analytical techniques used for microplastics’ qualification are affected by limitations, mainly related to microplastics’ size [67]. µ-FTIR showed a precise size resolution of >20 µm. Such size limitations are due to the limited resolution of IR diffraction. When microplastics smaller than 20 µm are analysed through µ-FTIR, the absorbance produced is not enough for obtaining interpretable spectra [67]. Microparticles of 3.8 [26], 10 [3] and 11 µm [71], extracted from drinking water and sea salt, were qualified using µ-FTIR. Similarly, microplastics of 5 µm, extracted from milk products, were confirmed as plastic via µ-FTIR [34], as well as for microplastics of 15.6 µm isolated from chicken meat [32]. Furthermore, 520 nm nanoplastics extracted from tea were qualified through µ-FTIR [56]. This should be reassessed based on the size limitation of the technique. It has been reported that for qualifying smaller micro (nano) plastics, µ-Raman seems to be more suitable. Microplastics smaller than 20 µm are likely undetectable using µ-FTIR.

Theoretically, µ-Raman could detect microplastics of 1 µm. However, such a limit is not decisive, as other parameters (i.e., complexity of the sample, filter type) could strongly influence the measurement [67].

### 4.4. Humans and Microplastics: Size and Impact on Humans

The experimental evidence available thus far is not sufficient to provide a comprehensive picture of the negative effects of microplastics on humans after ingestion. A work by Bouwmeester et al. (2015) [75] suggested that micro- and nano-plastics could be easily adsorbed by human tissues. Furthermore, penetration into organs’ barriers such as the brain has been suggested as possible, leading to harmful effects on the central nervous system [75]. However, the accumulation of microplastics should be considered as cell’s uptake. The accumulation/uptake by human cells (firstly by the human gastro intestinal tract if supposing an exposure via foods) is the essential factor to assess whether microplastics are a physical threat [76]. Both in vivo and in vitro experiments proved that cells’ microplastics uptake depends on microplastics’ size [77]. Therefore, negative and adverse effects on the human body are undoubtedly related to microplastics being “small enough” which can easily be internalised into cells [78]. The internalisation of microplastics mainly occurs through macrophages which performed phagocytosis and enterocytes (i.e., transcytosis). However, it has also been suggested that cells not specialized in phagocytosis (i.e., human intestine epithelial cells HT29) could be able to perform it [78]. After an oral ingestion of microplastics, they have to cross the intestinal mucosa to be adsorbed. Hence, transcytosis via enterocytes can be performed by internalization or they might be uptaken via macrophages (i.e., phagocytosis). Microplastics could be internalized by macrophages in the so-called Peyer Patches tissue and may be released to the blood and/or lymphatic circulation for translocation [1,18,79]. The microplastics range which may cross biological barriers is still not clear and definitive [80]. Thus far, microplastics of around 200 nm could be able to cross biological barriers and to be internalized into cellular systems. Further, only microplastics smaller than 200 nm could enter the blood circulation [66]. Such knowledge seems to confirm that microplastics’ sizes detected in food matrices analysed in this review are not biologically able to be embedded in human cells and thus to create adverse and harmful effects.

## 5. Conclusions

Microplastics’ occurrence in food products has been suggested from the scientific community. However, it has emerged that the standardization of analytical approaches is still far from being achieved. Sample preparations, in particular for matrices rich in terms of organic matter, affects the qualification of microplastics and, most importantly, the assessment of their size. Likewise, some papers included in this review showed discrepancies with the analytical methods (and instrumentation) used for the assessment of the plastic nature of microparticles isolated from foods. The selection of the methodological approach should take into account its size limitations; otherwise, artefact results could occur. Further, size is one of the most relevant parameters of microplastics for the assessment of the human health implications after ingestion. However, it could be suggested that the microplastics found in the studies included are “too big” for being accumulated into the human body. After ingestion, bigger microplastics could be excreted from the organism.

Therefore, the following could be suggested: (i) standardization of sample preparation (the type of digestion agent, time/temperature) based on the chemical reaction which occurs between the substrate (i.e., food) and the digestion agent; (ii) selection of the methodological approach for chemical analyses strongly considering the size limitations of the technique; (iii) accumulation phenomena into the human body could occur for nanoplastics rather than microplastics. Therefore, the optimisation of the analytical approach should be towards the isolation and quali-quantification of particles which can most likely accumulate into human body.

## Figures and Tables

**Table 1:** Search terms used for literature review. * indicates that both singular and plural terms were considered in the search.

Concept #1	Concept #2	Concept #3	Concept #4
Microplastic * plastic particle * microplastic particle *	Analytical determination * Size Sample treatment *	Human health Negative effect * Adverse effect *	Food Food matrices Beverages
OR	OR	OR	OR
AND			

**Table 2:** Experimental parameters used for digesting food organic matters of some studies included in this review. Only studies which focused on sample preparation were reported. N/A means that information was not available.

Digestion Agent	Time (h)	Temperature (°C)	Digestion Efficiency	Ref.
**Edible fish tissues**				
KOH (10%) + H_2_O_2_ (30%)	42	N/A	97.4 ± 0.5%	[10]
KOH (10%)	24	60	N/A	[59]
	36	60	N/A	[51]
HNO_3_ (6.3%)	Heating time of 10 min.	Up to 200	N/A	[11]
	Holding time of 10 min.	200		
HNO_3_ (69%)	72	60	98.55 ± 0.37%	[52]
H_2_O_2_ (30%)	24	30	N/A	[60]
	48	65	N/A	[44]
	24–48	R.T.		
	24	65	N/A	[48]
	24–48	R.T.		
	72	45	N/A	[12]
	72	60	74.73 ± 0.77%	[52]
enzymes +H_2_O_2_ (30%)	36	60	99–100%	[56]
NaOH (10%)	72	60	63.86 ± 1.47%	[52]
Sodium dodecyl sulfate (10%)	72	60	40.30 ± 2.70%	[52]
Trypsin (5%)	72	40	57.60 ± 7.85%	[52]
**Meat**				
KOH (10%)	10	75	N/A	[32]
	36	55		[57]
**Sea Salt**				
Not digested (dissolved into distilled water)	N/A	N/A	N/A	[17,18,19,28]
Fenton’s reagent	30 to 60 min	R.T and 75	N/A	[21]
H_2_O_2_ (30%)	24	65	N/A	[20,24]
	24	60		[22]
	24	50		[61]
	48	40		[23]
KOH (30%)	N/A	N/A	N/A	[25]
KOH (30%) + H_2_O_2_ (35%)	N/A	N/A	N/A	[25]
**Sugar**				
Not digested (dissolved into distilled water)	N/A	N/A	N/A	[18]
**Beer**				
H_2_O_2_	72	N/A	N/A	[16]
direct filtration	N/A	N/A	N/A	[29]
**Honey**				
H_2_O_2_	72	N/A	N/A	[16]
Milk				
KOH	48–72	N/A	N/A	[34]
**Lettuce**				
H_2_O_2_	N/A	N/A	N/A	[62]

**Table 3:** Size limitation of the techniques, microplastics’ size and type of polymers mostly used in the studies included.

Technique	Size Limitation of the Technique	Microplastics Size Range	Particle Type	Ref.
SEM-EDX	N/A	1.28–4.2 μm	N/A	[1]
LDIR	20–500 μm	6–480 μm	PET, PP, PA, PE	[2]
μ-FTIR	>20 μm	11–530 μm	PE	[3]
μ-ATR-FTIR	>7 μm	11–50 μm		
μ-Raman	>0.45 μm	~5 μm	PET	[4]
μ-FTIR	>20 μm	5–4659 μm	PET, PS, Nylon	[5]
μ-Raman	>0.45 μm	0.06–5.89 mm	PO	[6]
μ-FTIR	>20 μm	N/A	LDPE, PET, PE, PS, AC	[7]
μ-FTIR	>20 μm	0.005–5 mm	PET, Rayon, PES, Nylon, PP, CP, PE	[9]
μ-Raman	>0.45 μm	412–648 μm	PP, PE, PS, PET	[10]
μ-Raman	>0.45 μm	190–3800 μm	PP, PET	[13]
μ-FTIR	>20 μm	2.48–6742.48 mm	PE, PP, PAM	[16]
μ-ATR-FTIR μ-Raman	>7 μm >0.45 μm	0–1000 μm	PP, PA, PE	[17]
μ-ATR-FTIR	>7 μm	45 μm–4.3 mm	PE, PP	[18]
μ-ATR-FTIR	>20 μm	N/A	PP, PE, PES	[19]
μ-FTIR	>20 μm	0.39–7.02 mm	PE, PP	[20]
μ-ATR-FTIR	>7 μm	65–2500 μm	LDPE, HDPE	[21]
μ-ATR-FTIR	>7 μm	300–5000 μm	PP, PE, PET, PS	[22]
μ-Raman	>0.45 μm	5 to >1000 μm	Rayon, PP, PES, PE	[23]
μ-ATR-FTIR	>7 μm	23.2 μm–3.9 mm	PA, PU	[24]
μ-FTIR	>20 μm	45–100 μm	HDPE, PP, PET, PS, PA	[25]
μ-FTIR	>20 μm	3.8 μm–5.2 mm	CP, PS, PA, PAR	[26]
SEM-EDX	N/A	100–500 μm	PE	[27]
μ-FTIR	>20 μm	30 μm–3.5 mm	PET, PP, PE	[28]
μ-FTIR	>20 μm	N/A	PET	[29]
μ-FTIR	>20 μm	<300 μm	PVC, PET, PTFE, HDPE, Nylon, ABS	[30]
μ-FTIR	>20 μm	15.6–1151.1 μm	PE	[32]
μ-FTIR	>20 μm	5–20 μm	PS, PP, PE	[34]
μ-Raman	>0.45 μm	<5 μm	PET	[35]
μ-ATR-FTIR	>7 μm	N/A	PES, PET, PS	[42]
μ-Raman	>0.45 μm	1.2–10 μm	PE, PET, PC	[43]
μ-FTIR	>20 μm	0.06–0.11 mm	PE, PP, PS, Nylon	[44]
μ-FTIR	>20 μm	0.2–22 mm	PE, PP	[46]
μ-Raman	>0.45 μm	15–1175 μm	PS	[47]
SEM/EDX	N/A	100–1000 μm	PET, PE	[50]
μ-Raman	>0.45 μm	<300 μm	PE, PP, PET	[54]
μ-FTIR	>20 μm	520 nm–270 μm	Nylon, PET	[56]
μ-FTIR	>20 μm	453–3885 μm	Nylon, PP, HDPE	[60]
μ-Raman	>0.45 μm	N/A	PE, PP, PS, PVC, PET	[58]
μ-Raman	>0.45 μm	0.1–3 mm	PA, PET, PAS	[63]
μ-ATR-FTIR	>7 μm	24–1670 μm	PES, ABS, EPM, nylon-6, CP, and viscose	[51]
μ-FTIR	>20 μm	<100 μm to 5000 μm	PP, PE	[52]
μ-FTIR	>20 μm	50–500 μm	PVC, LDPE, PS, PP	[57]
μ-FTIR	>20 μm	20–150 μm	PE, PP, PS	[61]
μ-Raman	>0.45 μm			

Abbreviations: Polyolefin, PO; Ethylene-propylene copolymer, EPM.

## Data Availability

All the data are in the paper.
